# NMR and GC-MS Based Metabolic Profiling and Free-Radical Scavenging Activities of *Cordyceps pruinosa* Mycelia Cultivated under Different Media and Light Conditions

**DOI:** 10.1371/journal.pone.0090823

**Published:** 2014-03-07

**Authors:** Taek-Joo Oh, Sun-Hee Hyun, Seul-Gi Lee, Young-Jin Chun, Gi-Ho Sung, Hyung-Kyoon Choi

**Affiliations:** 1 College of Pharmacy, Chung-Ang University, Seoul, Republic of Korea; 2 Mushroom Research Division, Department of Herbal Crop Research, National Institute of Horticultural & Herbal Science, RDA, Eumseong, Republic of Korea; National Research Council of Italy, Italy

## Abstract

Variation of metabolic profiles in *Cordyceps pruinosa* mycelia cultivated under various media and light conditions was investigated using ^1^H nuclear magnetic resonance (NMR) analysis and gas chromatography mass spectrometry (GC-MS) with multivariate statistical analysis. A total of 71 metabolites were identified (5 alcohols, 21 amino acids, 15 organic acids, 4 purines, 3 pyrimidines, 7 sugars, 11 fatty acids, and 5 other metabolites) by NMR and GC-MS analysis. The mycelia grown in nitrogen media and under dark conditions showed the lowest growth and ergosterol levels, essential to a functional fungal cell membrane; these mycelia, however, had the highest levels of putrescine, which is involved in abiotic stress tolerance. In contrast, mycelia cultivated in sabouraud dextrose agar with yeast extract (SDAY) media and under light conditions contained relatively higher levels of fatty acids, including valeric acid, stearic acid, lignoceric acid, myristic acid, oleic acid, palmitoleic acid, hepadecenoic acid, and linoleic acid. These mycelia also had the highest phenolic content and antioxidant activity, and did not exhibit growth retardation due to enhanced asexual development caused by higher levels of linoleic acid. Therefore, we suggested that a light-enriched environment with SDAY media was more optimal than dark condition for cultivation of *C. pruinosa* mycelia as biopharmaceutical or nutraceutical resources.

## Introduction

The anamorph of *Cordyceps pruinosa* is the fungus *Mariannaea pruinosa*. *C. pruinosa*, which belongs to the Ascomycota phylum, is a well known entomogenous fungus that parasitizes the larvae of Lepidopter [1], [2]. Many beneficial genetic and biological characteristics of *C. pruinosa* mycelia have been identified. Among these are the many secondary metabolic compounds such as polysaccharides, N-6-(2-hydroxyethyl) adenosine, adenosine, and anti-ultraviolet radiation constituents [3]–[5].

Previous studies have shown that the methanol extract of the fruit body of *C. pruinosa* is able to curb NF-κB–dependent inflammatory gene expression, and that the mycelium of *C. pruinosa* improves cellular immune functions [6], [7]. Furthermore, the butanol fraction of *C. pruinosa* might play an effective role as an anti-proliferative agent for cancer therapy [8].

However, global metabolite profiling of *C. pruinosa* has not yet been performed. Metabolic profiling has the potential to help improve our understanding of the variety of physiological properties associated with diverse metabolites of *C. pruinosa.* However, it is known that metabolite production is affected by environmental factors such as light exposure and culture medium components. Many previous studies have investigated the effects of environmental changes on the production of secondary metabolites in *Cordyceps* species [9]–[12]. In addition, there have been many studies of antioxidants from other *Cordyceps* species, including *C. militaris* and *C. sinensis* [13], [14]. Until recently, metabolic profiles and antioxidative activity in *C. pruinosa* mycelium had not been investigated.

We hypothesized that the metabolic profile and antioxidative activity of *C. pruinosa* mycelia could be modulated by controlling cultivation conditions. The main objective of this study was to perform metabolic profiling in mycelia of *C. pruinosa* cultivated under various culture environments and determine the optimal culture conditions for the production of the mycelia with the highest antioxidant activity.

## Materials and Methods

### Solvents and chemicals

First grade methanol, hexane, water, D_2_O [99.%, containing 0.05% 3-(trimethylsilyl)-propionic-2,2,3,3-d4 acid sodium salt (TSP) as internal standard for NMR], methoxylamine hydrochloride, pyridine, L-ascorbic acid, 1,1-diphenyl-2-picrylhydrzyl (DPPH), Folin-Ciocalteu's phenol reagent, gallic acid, and sodium carbonate were purchased from Sigma (St. Louis, MO, USA). NaOD were obtained from Cortec (Paris, France). BSTFA [N,O-bis (trimethylsilyl) trifluoroacetamide containing 1% TMCS (trimethyl chlorosilane)] were purchased from Alfa Aesar (Ward Hill, MA, USA) and 2-Chloronaphtahalene as internal standard for GC-MS were purchased from Tokyo Chemical Industry Co., Ltd (Tokyo, Japan).

### Culture conditions for *C. pruinosa* mycelia

*C. pruinosa* ascospores discharged from fresh stroma were purchased from Mushtech Co. (Chuncheon, Korea). Culture materials including dextrose, agar, peptone, and yeast extract (Difco, Detroit, MI, USA) were purchased from Sigma (Sigma, St. Louis, MO, USA), and iron was purchased from Junsei Chemical (Junsei Chemical Co., Ltd, Japan). The ascospores were inoculated with SDY medium (cultivation conditions and media components are shown in Table 1) and incubated at 25°C. After storage for 2 days, 300 µL of the discharged ascospore were suspended in sterilized water and cultivated in the following media: sabouraud dextrose agar with yeast extract (SDAY) medium without light (S+D), SDAY medium with light (S+L), nut medium without light (L+D), nut with SDAY medium without light (SL+D), iron-supplemented SDAY medium without light (SF+D), and nitrogen-supplement medium without light (N+D).

**Table 1:** Composition of growth medium and light conditions for cultivation of *C. pruinosa* mycelia.

Medium	Medium composition (g/L)													Light condition	Abbreviation
	Dextrose	Peptone	Yeast extract	Iron- EDTA	Walnut powder	Peanut powder	Pine nut powder	Macro- elements*	Micro- elements**	FeCl_3_ •6H_2_O	amino acid***	yeast nitrogen	Agar	Dark /light	
SDAY	20	5	5	–	–	–	–	–	–	–	–	–	15	Dark	S+D
SDAY	20	5	5	–	–	–	–	–	–	–	–	–	15	Light	S+L
Nut	–	–	–	–	3.4	3.4	3.4	–	–	–	–	–	15	Dark	L+D
SDAY- Nut	20	5	5	–	3.4	3.4	3.4	–	–	–	–	–	15	Dark	SL+D
SDAY -Fe	20	5	5	0.04	–	–	–	–	–	–	–	–	30	Dark	SF+D
Nitrogen	–	–	–	–	–	–	–	100 (ml)	100 (ml)	1 (ml)	10 (ml)	6.7	15	Dark	N+D

*KH_2_PO_4_ 10 g/L, MgSO_4_ 7H_2_O 5 g/L, CaCl_2_ 2H_2_O 1 g/L. **CuSO_4_ 5H_2_O 0.04 mg/100 mL, BO_3_H_3_ 0.5 mg/100 mL, ZnSO_4_ 7H_2_O 0.4 mg/100 mL, MnSO_4_ H_2_O 0.4 mg/100 mL, NaMoO_4_ 2H_2_O 0.2 mg/100 mL. ***0.005% amino acid (L-glutamic acid, L-methionine, L-lysine, L-leucine, L-isoleucine).

To clearly separate cultivated mycelia from media at the harvest period, we used sterilized cellophane (583 Gel Dryer, Bio-SDAY medium Rad, Hercules, CA, USA) on the individual medium. All cultivation plates were incubated for 3 days at 25°C under either light (3,000 lux) or dark conditions, after which mycelia were collected from the cellophane. The mycelia were then lyophilized (FDU-1200, EYELA, Miyagi, Japan) for 24 h and frozen at −80°C until further analysis.

### NMR analysis

KH_2_PO_4_ (1.232 g) was added to 100 mL of D_2_O for NMR measurements as a buffering agent; 0.05% 3-(trimethylsilyl)-propionic-2,2,3,3-d4 acid sodium salt (TSP) was used as the internal standard for D_2_O. The pH of D_2_O was adjusted to 6.0 with the addition of 520 µL NaOD. The lyophilized powders of the mycelia from individual *C. pruinosa* samples (30 mg from each culture condition) were then extracted with 1 mL of D_2_O to obtain metabolic profiles of water-soluble metabolites. After sonication for 40 min, the samples were centrifuged at 2,000 rpm for 10 min (1730MR; Gyrogen Ltd., Daejeon, Korea), and then the supernatant was collected from each sample and filtered using a 0.45 µm filter (0.45 µm PTFE syringe filter for D_2_O). Each filtered sample (600 µL) was then transferred to a 5 mm NMR tube for analysis.

^1^H-NMR spectra were recorded at a temperature of 300 K on a 600.13-MHz Bruker Avance spectrometer (Bruker Analytische GmbH, Rheinstetten, Germany) using a cryoprobe. To suppress the residual water signal, we applied a zgpr pulse sequence. In total, 128 transients were gathered into 32 K data points with a relaxation delay of 2 s. An acquisition time per scan of 1.70 s and a spectral width of 9615.4 Hz were used. Before Fourier transformation, an exponential line broadening function of 0.30 Hz was applied to the free induction decay. In addition, 2D ^1^H-^13^C heteronuclear single quantum correlation (HSQC) experiments were additionally performed to confirm the assignment of metabolites. The HSQC spectra were obtained with a 2.0 s relaxation delay, 32 scans, and 5,896.2 Hz spectral width in F_2_ and 30, 864.2 Hz in F_1_.

MestReNova (version 6.0.4; Mestrelab Research SL, Santiago de Compostela, Spain) was used to obtain the NMR spectra, which were automatically encoded in ASCII files using AMIX (version 3.7; Bruker BioSpin, Billerica, MA, USA) software. The spectral ^1^H-NMR region from d = 0.56 to d = 10.00 was separated into regions with widths of 0.04 ppm, rendering 236 integrated regions in each NMR spectrum. ^1^H-NMR signal assignments were obtained by comparing their chemical shift with splitting patterns using the Chenomx NMR suite software (version 5.1; Chenomx, Edmonton, Canada). Each spectral intensity dataset was normalized to the total sum of the spectral regions.

### GC-MS analysis

To obtain separate extractions of non-polar and polar metabolites, both methanol and *n*-hexane were used as extraction solvents. Each sample (20 mg) cultivated under different conditions was put into a glass eppendorf tube (Axygen, Union City, CA, USA), and then extracted with 1 mL of 70% methanol and 100% *n*-hexane. Following sonication, the tube containing the extract was centrifuged at 2,000 rpm for 10 min. The supernatant was collected from each sample and filtered through a 0.45 µm filter (Acrodisc Syringe Filters, Pall Corporation, NY, USA). After extracting the samples, 100 µL of each sample solution was transferred into a GC vial to conduct derivatization and GC-MS analysis.

The solutions in GC vials were dried with a nitrogen gas flow for 5 min at 60°C, and then 30 µL of 20,000 µg/mL methoxylamine hydrochloride in pyridine for oximation, 50 µL of BSTFA (N,O-Bis (trimethylsilyl) trifluoroacetamide; Alfa Aesar, Ward Hill, MA, USA) containing 1% TMCS (trimethyl chlorosilane) for trimethylsilylation (TMS) derivatization, and 10 µL of 2-chloronaphthalene (Tokyo Chemical Industry Co., Ltd., Tokyo, Japan; 250 µg/mL in pyridine as an internal standard) were separately added to the dried samples. The derivatized samples were stored at 60°C for 60 min, and then subjected to GC-MS analysis.

To analyze the samples, a 7890A Agilent GC (Agilent Technologies, CA, USA) model equipped with a 5975C MSD detector (Agilent Technologies), autosampler (7683 B series, Agilent Technologies), split/splitless injector, injection module, and Chemstation software were used. Before analyzing the sample, the GC inlet temperature was set to 250°C with an injection volume of 1.0 µL and a split ratio of 1∶10, using helium as a carrier gas in constant-flow mode of 1.0 mL/min. The column used for analysis was a fused silica capillary column of 5% phenyl methylpolysiloxane phase (DB-5, Agilent Technologies) with dimensions of 30 m×0.25 mm i.d. ×0.25 µm film thickness.

Electron impact (EI) ionization mode was used for ionization. The detector voltage was 1400 V. The aux temperature, MS source temperature, and MS quad temperature were set to 280°C, 230°C, and 150°C, respectively. The mass range was 50–700 Da, and data were gathered in full scan mode.

For the polar metabolite analysis, The oven temperature was 80°C and was programmed to increase to 130°C (at 3°C/min) and then to 240°C (at 5°C/min) and then to 320°C (at 10°C/min; hold 3 min). For the non-polar metabolite analysis, The oven temperature was 80°C and was programmed to increase to 260°C (at 5°C/min) and then to 300°C (at 10°C/min; hold 3 min).

The mass spectra of the compounds were accepted when the match quality was more than 70% compared to the NIST-Wiley Mass Spectra Library for identification. It is important to obtain the raw GC-MS data to quantitatively compare metabolic profiles among all samples as described previously [15]. We used the Automated Mass Spectral Deconvolution and Identification System (AMDIS; http://chemdata.nist.gov/mass-spc/amdis/) for mass spectral deconvolution, which distinguishes peaks from noise and overlapping peaks. The setting values were as follows: component width = 12, adjacent peak subtraction = 1, medium resolution, medium sensitivity, and medium shape requirement. After finishing the program, ELU and FIN files were obtained as the output files of deconvolution and peak picking. However, the ELU files needed to be further analyzed with an online peak-filtering algorithm (SpectConnect, http://spectconnect.mit.edu) due to scatter and a tendency of the AMDIS data in multiple datasets to include false positives. The identification was performed using the spectra of each component, which were transferred to the NIST mass spectral search program MS Search 2.0, where they were matched to entries in the NIST MS library. The criterion for peak assignment was adopted when a match quality value was higher than 70%. Normalization to an internal standard peak area was used before multivariate statistical analysis. The relative intensities of assigned metabolites using GC-MS analysis were obtained.

### Statistical analysis

The results were evaluated by SIMCA-P software (version 13.0, Umetrics, Umeå, Sweden) for principal component analysis (PCA) and partial least squares regression (PLSR) was performed using mean-centered and unit variance scaled data. Clear differences in the content of the metabolites were detected by one-way analysis of variance (ANOVA) using IBM SPSS Statistics 19 software (IBM, Somers, NY), followed by the Tukey's significant-difference test. The level of statistical significance was set at *p*<0.05.

### DPPH assay

The latent antioxidant activity of each extract was evaluated based on the scavenging ability of the stable 1,1-diphenyl-2-picrylhydrazyl (DPPH) free radical, as described in a previous publication [16]. With a test tube, all sample solutions were added to separate wells (0.2 mL per sample), and then 3.8 mL of 100 mM DPPH was added to each tube. The tube was kept at room temperature in the dark for 30 min, and then the solution was transferred to a 96-well plate; the absorbance of the sample was measured at 520 nm. Blank solutions were prepared with 0.2 µL methanol and 3.8 µL DPPH. The antioxidant ability of each sample was calculated using the following formula: (inhibition rate, %)  =  (blank OD – sample OD)/blank OD ×100. Ascorbic acid was used as the positive control.

### Total phenolic content

The total phenolic content (TPC) in *C. pruinosa* cultivated under various conditions was measured using a modified version of the Folin-Ciocalteu method with gallic acid as the standard [17]. Each extract (0.2 mL) was mixed with 4.8 mL of distilled water, followed by addition of 0.5 ml of Folin-Ciocalteu's phenol reagent (Sigma). After incubating the mixture for 2 min at room temperature, 1.5 mL of 20% (w/v) sodium carbonate solution was added. The mixture was allowed to react for 2 h at room temperature, and its absorbance was then measured at 765 nm using a microplate spectrophotometer. The results were estimated using a calibration curve of gallic acid in the concentration range of 100–500 ppm; the results are expressed as mg of gallic acid equivalents (GAE) per dried extract.

## Results and Discussion

### Morphological characteristics of *C. pruinosa* mycelia

**Figure 1:**
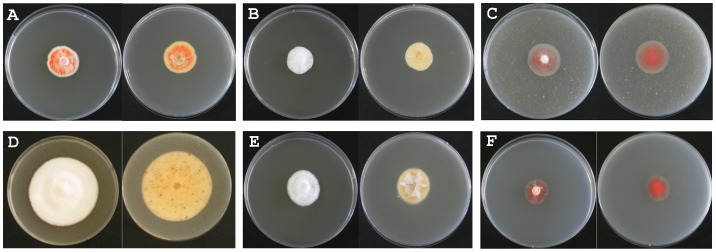
Morphological characteristics of *C. pruinosa* mycelia cultivated under various conditions. Left photo in each condition: front of plate. Right photo in each condition: back of plate. (A) S+L condition; (B) S+D condition; (C) L+D condition; (D) SL+D condition; (E) SF+D condition, (F) N+D condition.

The nutritional and cultural requirements of *Cordyceps* species have been studied for many years with the aim of cultivating them under artificial conditions, from which nutritionally rich SDAY medium has been determined to be the most optimal medium [18]. *C. pruinosa* mycelia cultivated in SDAY medium are shown in Figure 1.

*C. pruinosa* is deep or light red due to a red pigment present in its cells. We grew the mycelia of *C. pruinosa* under light and dark conditions, adding lipid, iron, and nitrogen individually to each mycelium under dark conditions. The following 6 culture conditions were used: dextrose agar supplemented with 0.5% SDAY medium without light (S+D), which were usually used as traditional cultivation conditions;, SDAY medium with light (S+L) as a control group; nut medium without light (L+D), which was used to measure lipid metabolism of the mycelium by adding unsaturated fatty acid to the medium; nut with SDAY medium without light (SL+D) to investigate the effect of complemented lipid nutrients on mycelial metabolism; iron-supplemented SDAY medium without light (SF+D) to analyze the effects of iron, known to be important in growth of *Cordyceps* specie [19]; and nitrogen medium without light (N+D) because of its role in the biosynthesis of alkaloids, including cordycepin and adenosine (Table 1).

Growth characteristics such as mycelia density, colony diameter, and pigmentation were similar in samples grown in the S+L and L+D conditions, which were different from the characteristics observed for the S+D and SF+D samples. In particular, mycelia density and colony diameter in the SL+D group were the highest compared to mycelia grown in the other conditions. Mycelia cultivated in the N+D condition grew more slowly than those cultivated under other conditions.

Mycelia grown under all conditions appeared white at the top of the colonies, whereas those at the bottom of the colonies exhibited the following differences. Mycelia cultivated under S+L, L+D, and N+D conditions were red, while those of S+D, SL+D, and SF+D appeared white. Morphological differences were also observed between mycelia cultivated under various conditions, implying that there were likely different metabolic profiles induced by different culture conditions.

### Metabolite assignment by NMR and GC-MS analysis

**Figure 2:**
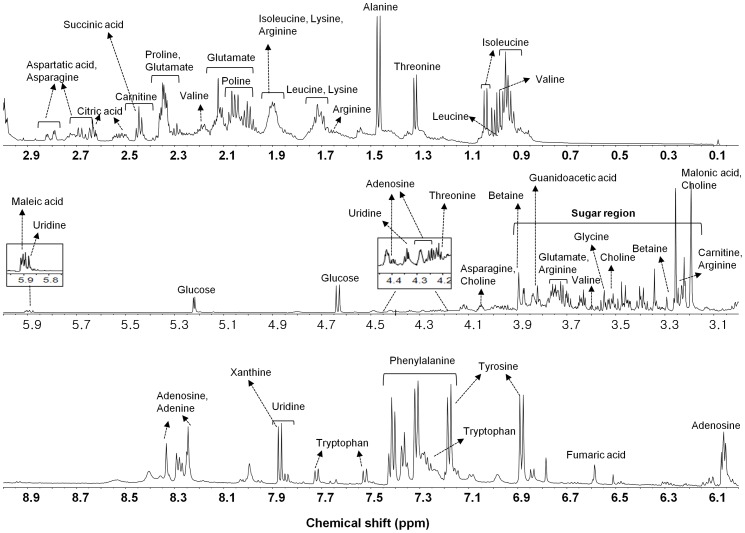
Representative ^1^H NMR spectra (600 MHz) of *C. pruinosa* mycelia extracts.

The D_2_O extracts of the *C. pruinosa* mycelia cultivated under various conditions were analyzed using NMR spectroscopy to characterize global metabolic profiles. A representative ^1^H NMR spectrum of the extracts obtained from *C. pruinosa* mycelia is shown in Figure 2. The metabolites were identified by comparing the chemical shifts of standard compounds using the NMR Suite software (version 5.1; Chenomx). As shown in Table 2, thirty-three metabolites were detected. ^1^H-^13^C HSQC analysis was performed to confirm the assignment, and fourteen compounds, such as isoleucine, leucine, valine, threonine, alanine, lysine, proline, asparagine, choline, glucose, betaine, glycerol, uridine and adenosine were confirmed by ^1^H-^13^C HSQC (Fig. S1A and Fig. S1B).

**Table 2:** Assignment of ^1^H NMR spectral peaks for *C. pruinosa* mycelia using D_2_O extracts.

No	Compound	Chemical shift (multiplicity, *J* value)
1	Isoleucine	0.93(t, *J* = 7.20), 1.03(d, *J* = 7.03), 1.21–1.29(m)
2	Leucine	0.95(t, *J* = 6.00), 1.64–1.76(m)
3	Valine	0.98(d, *J* = 6.96), 1.03(d, *J* = 6.97), 2.15–2.22(m), 3.60 (d, *J* = 426)
4	Threonine	1.32(d, *J* = 6.6), 3.60(d, *J* = 4.98), 4.22–4.28(m)
5	Alanine	1.47(d, *J* = 7.2), 3.74–3.80(m)
6	Arginine	1.65–1.77(m), 1.85–1.96(m), 3.21–3.25(m), 3.70(t, *J* = 6.18)
7	Lysine	1.67–1.76(m), 1.83–1.95(m), 3.01(t, *J* = 7.68)
8	Proline	1.94–2.09(m), 2.30–2.37(m), 4.10–4.14(m)
9	Glutamate	2.00–2.10(m), 2.10–2.16(m), 2.29–2.40(m), 3.76(dd, *J*_1_ = 6.2, *J*_2_ = 3.8)
10	Carnitine	2.41–2.48(m), 3.22(s)
11	Succinic acid	2.44(s)
12	Citric acid	2.53(d, *J* = 16.2), 2.64 (d, *J* = 16.56)
13	Asparagine	2.67(dd, *J*_1_ = 12, *J*_2_ = 7.74), 2.80(dd, *J*_1_ = 18, *J*_2_ = 4.32)
14	Aspartatic acid	2.68(dd, *J*_1_ = 18.3, *J*_2_ = 9.5), 2.82(dd, *J*_1_ = 17.5, *J*_2_ = 3.6),
15	Choline	3.19(s), 3.49–3.53(m), 4.03–4.08(m),
16	Malonic acid	3.20(s)
17	Glucose	3.21–3.25(m), 3.37–3.42(m), 3.43–3.48(m), 3.50–3.55(m), 3.67–3.78(m), 3.80–3.85(m), 3.88(dd, *J*_1_ = 12.18, *J*_2_ = 2.10), 4.63(d, *J* = 7.95), 5.22(d, *J* = 3.74)
18	Betaine	3.26(s), 3.90(s)
19	Glycerol	3.52–3.57(m), 3.66(dd, *J*_1_ = 12, *J*_2_ = 4.32)
20	Glycine	3.55(s)
21	Glucitol	3.61–3.67(m), 3.72–3.79(m), 3.80–3.86(m)
22	Guanidoacetic acid	3.82(s)
23	Uridine	4.21(t, *J* = 5.52), 4.34(t, *J* = 5.1), 5.87–5.92(m), 7.87(d, *J* = 8.13)
24	Adenosine	4.27–4.30(m), 4.38–4.40(m), 6.06(d, *J* = 6.12), 8.24(s), 8.33(s)
25	Tartric acid	4.34(s)
26	Maleic acid	5.90(s)
27	Fumaric acid	6.59(s)
28	Tyrosine	6.89(d, *J* = 8.52), 7.16–7.20(m)
29	Tryptophan	7.19(t, *J* = 7.23), 7.25–7.30(m), 7.53(d, *J* = 8.24), 7.72(d, *J* = 9.53)
30	Phenylalanine	7.30–7.33(m), 7.34–7.38(m), 7.39–7.44(m)
31	Phenylacetic acid	7.25–7.33(m), 7.34–7.39(m)
32	Xanthine	7.87(s)
33	Adenine	8.24(s), 8.33(s)

To maximize the number of metabolites that could be investigated, GC-MS was employed to perform global metabolite profiling. There were 44 identified compounds in the 70% methanol extracts and 14 identified compounds in the 100% *n*-hexane extracts. The detected metabolites could be divided into several classes, including alcohols, amino acids, organic acids, purines, sugars, fatty acids, sterols, and pyrimidines (Table 3 and Table 4). The relative levels of various metabolites in the 70% methanol extracts and 100% *n*-hexane extracts are presented in Table S1 and Table S2.

**Table 3:** Chromatographic data of the identified compounds from the 70% MeOH extract of *C. pruinosa* mycelia analyzed using GC-MS.

Identified compounds	RT	MS fragment ion (m/z)	TMS
**Alcohols**			
Glucitol	39.14	387, 357, 299, 217, 147, 73	6TMS
Glycerol	13.14	205, 147, 117, 103, 73	3TMS
Myo- inositol	32.03	318, 217, 191, 147, 73	6TMS
	33.46	318, 217, 191, 147, 73	6TMS
	34.61	318, 217, 191, 147, 73	6TMS
	34.75	318, 217, 191, 147, 73	6TMS
	40.05	318, 217, 191, 147, 73	6TMS
	31.53	319, 205, 147, 103, 73	6TMS
Ribo-hexitol	29.56	231, 205, 147, 129, 73	6TMS
Xylitol	26.71	307, 217, 205, 147, 103, 73	5TMS
	27.05	307, 217, 205, 147, 103, 73	5TMS
**Amine**			
Putrescine	27.35	361, 214, 200, 174, 86	4TMS
**Amino acids**			
Alanine	18.93	290, 248, 174, 147, 73	3TMS
Asparagine	25.86	231, 188, 147, 132, 116, 73	3TMS
Aspartic acid	21.95	232, 218, 147, 100, 73	3TMS
Cystathionine	36.86	278, 218, 128, 73	4TMS
Glutamine	24.69	348, 246, 147, 128, 73	TMS
	28.32	347, 245, 156, 73	3TMS
Glycine	14.25	276, 248, 174, 147, 73	TMS
	20.31	320, 218, 174, 147, 73	3TMS
Histidine	31.33	254, 182, 154, 73	3TMS
Homoserine	19.72	320, 218, 147, 128, 103, 73	3TMS
Isoleucine	13.82	218, 158, 147, 73	2TMS
Lysine	26.80	362, 156, 102, 84, 73	3TMS
	31.47	317, 230, 174, 156, 128, 73	4TMS
Ornithine	29.22	420, 200, 174, 142, 73	4TMS
Proline	13.96	216, 142, 73	2TMS
	21.79	258, 230, 156, 147, 73	2TMS
	22.04	230, 140, 73	3TMS
Serine	12.44	219, 147, 132, 116, 103, 73	2TMS
	16.49	278, 218, 204, 147, 100, 73	3TMS
Threonine	17.46	291, 218, 147, 117, 101, 73	3TMS
Tyrosine	31.74	382, 280, 218, 179, 147, 73	3TMS
Valine	10.91	218, 144, 73	2TMS
**Organic acids**			
Acetic acid	19.21	263, 204, 177, 147, 117, 73	2TMS
Citric acid	29.35	465, 363, 347, 273, 147, 73	4TMS
Fumaric acid	16.18	245, 147, 73	2TMS
Galactonic acid	32.82	433, 319, 292, 205, 147, 73	6TMS
Gluconic acid	32.90	333, 319, 292, 205, 147, 73	6TMS
	40.16	387, 357, 333, 299, 147, 73	6TMS
Glucuronic acid	39.58	449, 305, 204, 217, 147, 73	6TMS
Glutaric acid	23.53	304, 288, 198, 147, 73	2TMS
Glyceric acid	15.29	292, 205, 189, 147, 133, 73	3TMS
Succinic acid	14.71	247, 147, 73	2TMS
	20.99	304, 174, 147, 86, 73	3TMS
γ-aminobutyric acid	9.41	204, 147, 130, 73	2TMS
	22.11	304, 174, 147, 86, 73	3TMS
**Purines**			
Adenine	30.22	279, 264, 192 84, 73	2TMS
Adenosine	42.65	540, 245, 236, 230, 217, 192	4TMS
	44.06	498, 236, 188, 175, 148, 73	4TMS
	46.15	692, 315, 258, 230, 169, 73	5TMS
Uric acid	34.87	456, 444, 384, 369, 147, 73	4TMS
Xanthine	33.31	368, 353, 294, 279, 147, 73	3TMS
**Pyrimidine**			
Cytidine	40.54	245, 217, 168, 151, 73	3TMS
**Sugars**			
Arabinose	29.13	217, 204, 191, 147,73	4TMS
Erythrose	21.57	307, 217, 205, 147, 103, 73	3TMS
Fructose	26.07	307, 217, 147, 103, 73	6TMS
	30.58	307, 217, 147, 103, 73	
Galactose	29.82	319, 217, 147, 133, 73	5TMS
	30.66	319, 217, 205, 160, 147, 73	6TMS
	31.00	217, 204, 191, 147, 73	6TMS
	39.44	217, 204, 191, 147, 73	6TMS
	41.19	319, 217, 205, 160, 147, 73	6TMS
Glucose	29.00	217, 204, 191, 147, 73	6TMS
	31.24	319, 205, 160, 147, 103, 73	6TMS
	32.57	435, 217, 204, 191, 147, 73	6TMS
	34.74	494, 259, 173, 147, 131, 73	5TMS
	36.80	364, 319, 217, 205, 160	5TMS
	39.74	387, 299, 204, 147, 129, 73	6TMS
	43.69	437, 361, 217, 73	8TMS
	44.78	387, 299, 204, 147, 129, 73	6TMS
Mannose	30.83	435, 217, 204, 191, 147, 73	6TMS
N-acetylglucosamine	33.86	245, 217, 191, 147, 73	6TMS
	35.10	245, 217, 191, 147, 73	6TMS

**Table 4:** Chromatographic data of the identified compounds from the 100% *n*-hexane extract of *C. pruinosa* mycelia analyzed using GC-MS.

Identified compounds	RT	MS fragment ion (m/z)	TMS
**Pyrimidine**			
Uracil	11.70	256, 241, 147, 99, 73, 45	2TMS
**Saturated fatty acids**			
Myristic acid	23.45	285, 145, 132, 117, 73, 55	TMS
Valeric acid	25.42	299, 145, 129, 117, 73, 55	TMS
Arachidic acid	33.72	367, 129, 117, 73, 55, 45	TMS
Lignoceric acid	39.30	425, 145, 132, 117, 73, 57	TMS
Margaric acid	29.12	327, 145, 132, 117, 73, 55	TMS
Palmitic acid	36.41	459, 371, 239, 203, 147, 73	2TMS
Stearic acid	30.89	341, 145, 132, 117, 73	TMS
**Unsaturated fatty acids**			
Heptadecenoic acid	28.67	325, 145, 129, 117, 73, 55	TMS
Linoleic acid	29.42	308, 263, 109, 87, 67, 55	TMS
	29.92	335, 129, 108, 95, 73, 55	TMS
	30.30	337, 262, 150, 129, 95, 75	TMS
	31.22	335, 129, 108, 95, 73, 55	TMS
	31.91	337, 262, 150, 129, 95, 75	TMS
Oleic acid	29.54	264, 222, 111, 97, 88, 55	TMS
	30.42	339, 145, 129, 117, 73, 55	TMS
	30.50	339, 145, 129, 117, 73, 55	TMS
	30.60	339, 145, 129, 117, 73, 55	TMS
	38.45	485, 397, 147, 129, 103, 73	TMS
Palmitoleic acid	26.91	311, 145, 129, 117, 73, 55	2TMS
**Sterols**			
Dehydroergosterol	43.66	470, 343, 255, 147, 107, 69	TMS
Ergosterol	43.41	468, 363, 337, 253, 143, 69	TMS

Significantly higher levels of γ-aminobutyric acid (GABA) were observed in mycelia from the SF+D group (Table S1). It has been reported that, compared to other mushrooms, *C.* mycelia are good sources of GABA [20]. As a result of *C.* mycelia grown under usual conditions in prior study, it seems that iron supplementary medium could stimulate and be optimal production of GABA. Compared to the S+D group, significant changes in lipid metabolism were not observed in mycelia grown under L+D and SL+D conditions (Table S2).

Compounds identified by NMR and GC-MS are presented in Table 5. The following 19 metabolites were observed from both NMR and GC-MS analysis: isoleucine, valine, threonine, alanine, lysine, proline, succinic acid, citric acid, asparagine, aspartic acid, glucose, glycerol, glycine, glucitol, adenosine, fumaric acid, tyrosine, xanthine, and adenine. 14 metabolites, such as phenylalanine, tryptophan, betaine, choline, and leucine were only observed using NMR. 39 metabolites, including inositol, putrescine, ornithine, γ-aminobutyric acid, uric acid, uracil, fatty acids, and ergosterol were only observed in the GC-MS analysis.

**Table 5:** The list of metabolites identified by ^1^H NMR and GC-MS analysis.

Analytical platform	Identified compounds
^1^H NMR	Leucine, arginine, carnitine, choline, malonic acid, betaine, uridine, guanidoacetic acid, tartaric acid, maleic acid, tryptophan, phenylalanine, phenylacetic acid
GC-MS	Inositol, ribo-hexitol, xylitol, putrescine, cystathionine, histidine, homoserine, ornithine, serine, acetic acid, galactonic acid, gluconic acid, glucuronic acid, glutaric acid, glyceric acid, γ-aminobutyric acid, uric acid, cytidine, arabinose, erythrose, fructose, galactose, mannose, N-acetylglucosamine, uracil, myristic acid, valeric acid, arachidic acid, lignoceric acid, margaric acid, palmitic acid, stearic acid, heptadecenoic acid, linoleic acid, oleic acid, palmitoleic acid, dehydroergosterol, ergosterol
^1^H NMR and GC-MS	Isoleucine, valine, threonine, alanine, lysine, proline, glutamic acid, succinic acid, citric acid, asparagine, aspartic acid, glucose, glycerol, glycine, glucitol, adenosine, fumaric acid, tyrosine, xanthine, adenine

### PCA based on NMR and GC-MS datasets

To provide comparative interpretations and visualization of metabolic changes under various culture conditions, PCA was applied to the NMR and GC-MS spectral datasets. PCA is a powerful tool to selectively identify the major controlling factors contributing to differences between samples without *a priori* knowledge of what those factors might be. PCA can allow for the identification of not only how a specific sample differs from other samples, but also what variables contributed to those differences [21].

**Figure 3:**
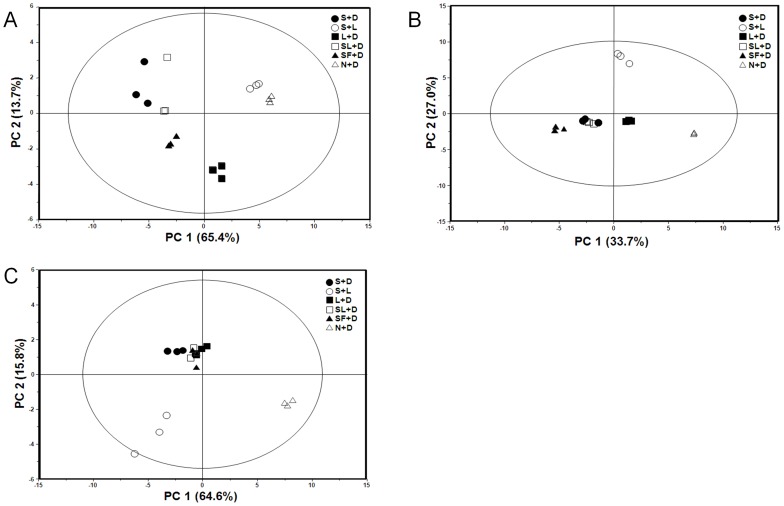
PCA score plots of *C. pruinosa* mycelia cultivated under various conditions. (A) D_2_O extracts characterized using NMR analysis. (B) 70% methanol extracts and (C) 100% *n*-hexane extracts characterized using GC-MS analysis. PCA, Principal component analysis.

The PCA score plot and loading plot of the D_2_O extracts analyzed by NMR are presented in Figure 3A and S2, respectively. In the score plots, PC 1 explained 65.4% of the variation and PC 2 explained 13.7% of the variation. S+D, SF+D, and SL+D were clearly distinct from the other conditions, S+L, N+D, and L+D along PC 1, while SF+D and L+D were separated from the others along PC 2. It was shown that relatively higher levels of glutamine, proline, alanine, arginine, and glucose were contained in S+D, SF+D, and SL+D, while those of isoleucine, leucine, tryptophan, tyrosine, adenosine, and phenylalanine were higher in S+L, N+D, and L+D from the loading plot analysis of D_2_O extracts (Fig. S2).

In Figure 3B and S3, the PCA score plot and loading plot for 70% methanol extracts determined using GC-MS are shown; PC 1 and PC 2 represented 33.7% and 27.0% of the variance across samples, respectively. The trend of the clusters was similar to that observed for the D_2_O extracts (Fig. 3A). S+D, SL+D, and SF+D were clearly distinguished from S+L, L+D, and N+D along PC 1, while S+L was completely separated from the others. From the loading plot analysis of 70% methanol extracts, relatively higher levels of aspartic acid, mannose, glucose, and γ-aminobutyric acid were observed ,in S+D, SF+D, and SL+D, whereas those of myo-inositol, putrescine, histidine, cystathionine, and adenine were observed in S+L, L+D, and N+D (Fig. S3).

The PCA score plot and loading plot for 100% *n*-hexane extracts analyzed by GC-MS are shown in Figure 3C and S4. PC 1 and PC 2 represented 64.6% and 15.8% of the sample variance, respectively. Unlike Figure 3A and 3B, S+D, SL+D, L+D, and SF+D were closely aggregated, whereas S+L and N+D were separated from the other samples. The loading plot analysis of 100% n-hexane extracts showed the lignoceric acid, linoleic acid, palmitoleic acid, oleic acid, and heptadecenoic acid were relatively abundant in S+L, and dehydroergosterol, margaric acid, and arachidic acid were relatively higher in N+D (Fig. S4).

Interestingly, there was a similar clustering tendency observed for both the NMR D_2_O extracts (Fig. 3A) and GC-MS 70% methanol extracts (Fig. 3B) based on the color of the bottom portions of the collected mycelia. The S+L, N+D, and L+D conditions, which had mycelia that were red in color, clustered in the PCA plots along PC 1, whereas the S+D, SL+D, and SF+D, which were white in color, were also clustered together in the score plots (Fig. 3A and 3B).

### Antioxidant activity

The free radical scavenging activities (FRSAs) of the 70% methanol extracts of *C. pruinosa* mycelia grown under various cultivation conditions are summarized in Table 6. The highest FRSAs were obtained from samples grown under the SL+D condition. The FRSA of the SL+D sample (64.08%) was similar to that of 0.2 mg/mL ascorbic acid (62.65%). The FRSAs from samples of the S+L and N+D conditions were similar to each other. Relatively lower FRSAs were observed in the samples from the S+D, L+D, and SF+D conditions.

**Table 6:** Antioxidant ability and total phenolic content of the 70% methanol extracts of *C. pruinosa* mycelium grown in different cultivation conditions.

Sample	Free radical scavenging activity (%) (50,000 mg/L)	TPC (GAE mg/g extract) (50,000 mg/L)
S+D	23.48±2.43^a^	2.95±0.26^a^
S+L	59.57±2.20^bc^	8.77±0.57^b^
L+D	38.21±0.72^d^	3.99±0.04^c^
SL+D	64.08±2.93^c^	5.87±0.27^d^
SF+D	11.74±0.95^e^	2.06±0.11^e^
N+D	57.56±1.58^b^	6.94±0.30^f^
Ascorbic acid (200 mg/L)	62.65±1.93^bc^	

GAE: gallic acid equivalents.

Cytotoxic effect of the mycelia was investigated by MTT assay and the cell viabilities in all extracts were over 80% in all conditions, indicating that there was no significant cytotoxicity of *C. pruinosa* mycelia extracts (data not shown).

Polyphenolic compounds are important constituents of mushrooms. Mushrooms contain various polyphenolic compounds that serve as excellent antioxidants due to their ability to scavenge free radicals [22]–[24]. The TPCs of *C. pruinosa* samples cultivated under various conditions are listed in Table 6. Samples from the S+L condition had the highest TPC, followed by samples from the N+D, SL+D, L+D, S+D, and SF+D conditions.

There was a strong positive linear relationship (correlation coefficient of 0.8058) between antioxidant activity and TPC for *C. pruinosa* mycelia extracts. This result suggests that the TPC contributed significantly to the antioxidant ability in *C. pruinosa* mycelia cultivated under various conditions.

Partial least squares regression (PLSR) was performed to evaluate the correlation between FRSAs and metabolites analyzed by NMR and GC-MS. As shown in Figure S5, the correlation efficient was R^2^ = 0.69. However, phenolic compounds known to contribute to antioxidant activity were not fully covered by NMR and GC-MS analysis in this study. Thus, LC-MS analysis will be employed to analyze metabolites including various phenolic compounds having FRSAs in further studies.

### Comparison of metabolic profiles

**Figure 4:**
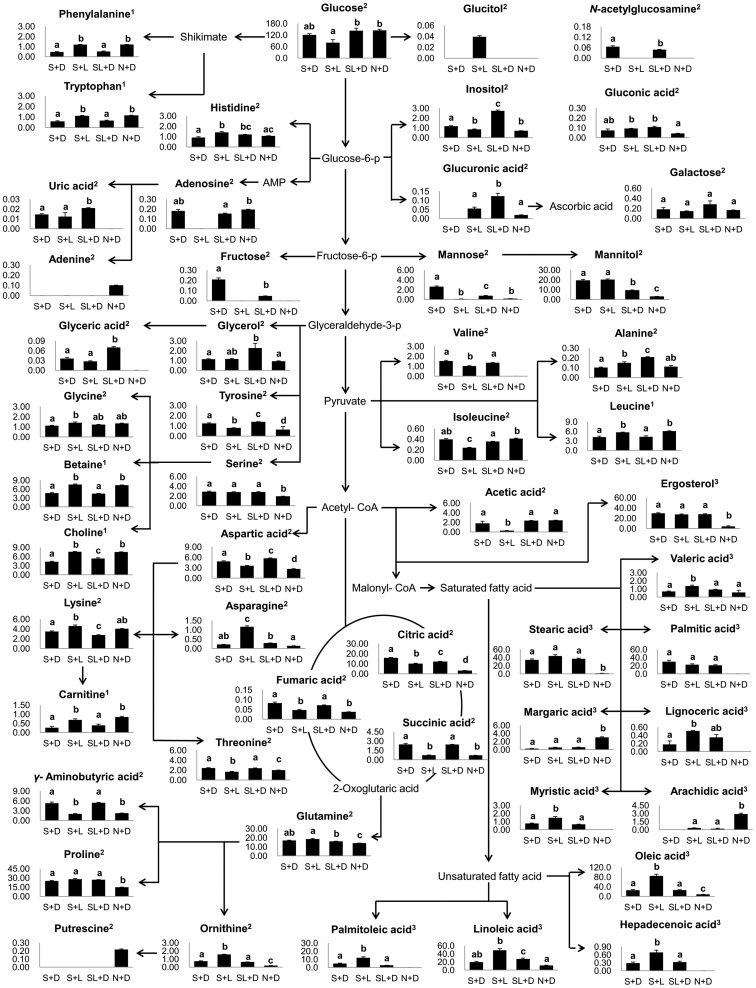
Schematic diagram of the metabolic pathway and relative levels of the major compounds detected in *C. pruinosa* extracted with 70% MeOH and 100% *n-*hexane plus D_2_O. This was modified from pathways presented in KEGG database (http://www.genome.jp/kegg/). ANOVA was performed to assess the statistical significance of differences between samples (p<0.05). Data are mean values with error bars representing standard deviation values. Different letters in bars represent the difference of statistical significance of metabolites levels. The superscript numbers in compounds represent analysis methods. 1: NMR analysis of D_2_O extracts, 2: GC-MS analysis of 70% methanol extracts, 3: GC-MS analysis of n-hexane extracts.

Our results showed that there were distinct differences in morphological, metabolic, and FRSA characteristics between *C. pruinosa* mycelia grown under different cultivation conditions. Metabolic profiles of S+L, N+D, and SL+D samples showing relatively higher antioxidant activities were further investigated and compared to S+D samples. This comparison is illustrated in Figure 4. The metabolic pathways were prepared by combining data from 5 metabolites identified using NMR and 47 metabolites identified by the GC-MS analysis.

The highest levels of adenosine, adenine, and carnitine were obtained in the N+D samples, but the mycelial growth was retarded in these samples. Putrescine, which was only detected in the N+D samples, is known to be one of the major polyamines involved in cell division and tolerance against abiotic stress in mushrooms [25]. The existence of putrescine only in the N+D samples may be due to an adaptation to an adverse environment for mycelia formation. Putrescine is synthesized from ornithine by ornithine decarboxylase [26]. We found that ornithine levels were the lowest in the N+D condition. The low ornithine levels in N+D samples might also be correlated with an increase in putrescine synthesis.

In addition, ergosterol is a specific component of the fungal cell membrane and is a precursor of vitamin D_2_ [27]. Similar levels of ergosterol were detected in samples grown under the S+L and SL+D conditions, but not under N+D condition. Given that ergosterol in *C. pruinosa* plays an important role in cell maintenance, the relatively lower ergosterol content in N+D samples indicates that this is an unsuitable condition for cell growth.

Levels of fatty acids, such as valeric acid (C5:0), myristic acid (C14:0), stearic acid (C18:0), lignoceric acid (C24:0), heptadecenoic acid (C17:1), linoleic acid (C18:2), oleic acid (C18:1), and palmitoleic acid (C16:1), were highest in samples grown under the S+L condition. However, sugar levels, including those of glucose, galactose, and mannose were lowest in S+L samples. Moreover, fructose and *N*-acetylglucosamine were not detected in the S+L condition.

Contrary to our results, it was previously reported that *Aspergillus ornatus* and *Blastocladiella emersonii* exposed to light showed growth retardation due to limited uptake of essential nutrients [28], [29]. *Aspergillus* species are known to develop either asexually in the light or sexually in the dark [30], [31]. Asexual spore development was also shown to be enhanced by linoleic acid [32], [33]. We suggest that enhanced linoleic acid in response to light might stimulate conidia formation in the context of asexual development in *C. pruinosa*. Thus, growth in samples cultivated under the S+L condition might not be retarded compared to that observed for S+D samples. The mycelia of *C. cardinalis*, *C. bassiana*, and *C. militaris* are commonly cultivated in SDAY agar medium in the dark to produce fruiting bodies [34]–[36]. However, our data suggest that the light condition is more optimal than dark condition for antioxidative activity, linoleic acid content, and mycelial growth in cultivation of *C. pruinosa* mycelia with SDAY medium.

## Conclusion

In this study, *C. pruinosa* mycelia cultivated under various media and light conditions showed differences in growth, metabolic profiles, and FRSAs. Higher levels of FRSAs were achieved in samples cultivated under SL+D, S+L, and N+D conditions. The mycelia grown under the S+L condition contained relatively higher levels of fatty acids, including linoleic acid, which is known for stimulating fungal conidia formation. Therefore, growth retardation was not observed in S+L samples because of enhanced asexual development caused by linoleic acid. Our finding revealed that *C. pruinosa* mycelia cultivated under the S+L (SDAY media and light condition) condition had higher antioxidative activity, linoleic acid content, and mycelial growth. To the best of our knowledge, this is the first report on effect of light on mycelial growth and metabolic profiling of *C. pruinosa*. Light was more beneficial condition than dark for antioxidative activity, linoleic acid content, and mycelial growth in cultivation of *C. pruinosa* mycelia with SDAY medium. This result can be applied to *C. pruinosa* fruiting body cultivation as a means of producing biopharmaceutical or natural medicinal resources.

## Supporting Information

Figure S1 **Representative ^1^H-^13^C HSQC NMR spectra of** ***Cordyceps pruinosa*** **mycelia by D_2_O extraction.** (a)^ 1^H chemical shift of 0–3 ppm, (b) ^1^H chemical shift of 3–6 ppm (1: isoleucine, 2: leucine, 3: valine, 4: threonine, 5: alanine, 7: lysine, 8: proline, 13: asparagine, 15: choline, 17: glucose, 18: betaine, 19: glycerol, 23: uridine, 24: adenosine).
(TIF)

Figure S2 **PCA loading plots derived from NMR analysis of D_2_O extracts of** ***C. pruinosa*** **mycelia cultivated under** **various conditions.**
(TIF)

Figure S3 **PCA loading plots derived from GC-MS analysis of 70% methanol extracts of** ***C. pruinosa*** **mycelia cultivated under various conditions.**
(TIF)

Figure S4 **PCA loading plots derived from GC-MS analysis of 100%** ***n*** **-hexane extracts of** ***C. pruinosa*** **mycelia cultivated under various conditions.**
(TIF)

Figure S5 **PLS t_1_/u_1_ score plots representing relationship between the free radical scavenging activities (u_1_) and metabolic profiles (t_1_) obtained by NMR and GC-MS.**
(TIF)

Table S1 **A GC-MS-based metabolic profile of 70% methanol extracts of** ***C. pruinosa*** **mycelia.** The relative levels of each metabolite were obtained by dividing the percentage area of each metabolite by the percentage area of the internal standard. Different letters in the same row indicate a significant difference. Mean±SD values for triplicate measurements are shown. ‘ND’ means ‘not detected’.
(DOCX)

Table S2 **A GC-MS-based metabolic profile of 100% n-hexane extracts of** ***C. pruinosa*** **mycelia.** The relative levels of each metabolite were obtained by dividing the percentage area of each metabolite by the percentage area of the internal standard. Different letters in the same row indicate a significant difference. Mean±SD values for triplicate measurements are shown. ‘ND’ means ‘not detected’.
(DOCX)
