# Supplementary figures and images for: NMR and GC-MS Based Metabolic Profiling and Free-Radical Scavenging Activities of Cordyceps pruinosa Mycelia Cultivated under Different Media and Light Conditions
Source: PLoS One. 2014 Mar 7;9(3):e90823. doi: 10.1371/journal.pone.0090823 (PMC3946585; doi:10.1371/journal.pone.0090823)

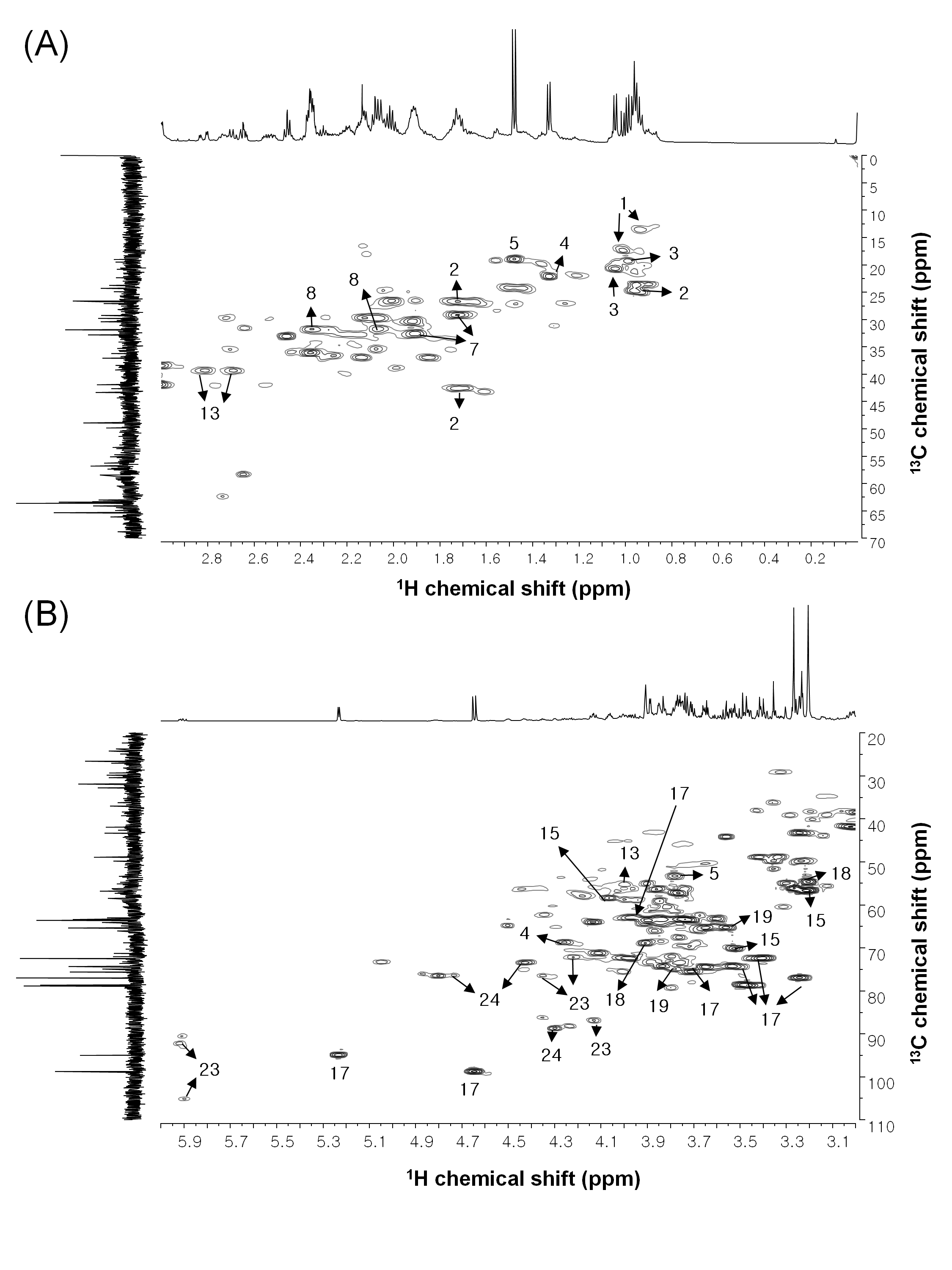

Supplement: Figure S1 — Representative 1H-13C HSQC NMR spectra of Cordyceps pruinosa mycelia by D2O extraction. (a) 1H chemical shift of 0–3 ppm, (b) 1H chemical shift of 3–6 ppm (1: isoleucine, 2: leucine, 3: valine, 4: threonine, 5: alanine, 7: lysine, 8: proline, 13: asparagine, 15: choline, 17: glucose, 18: betaine, 19: glycerol, 23: uridine, 24: adenosine). (TIF) [file pone.0090823.s001.tif]

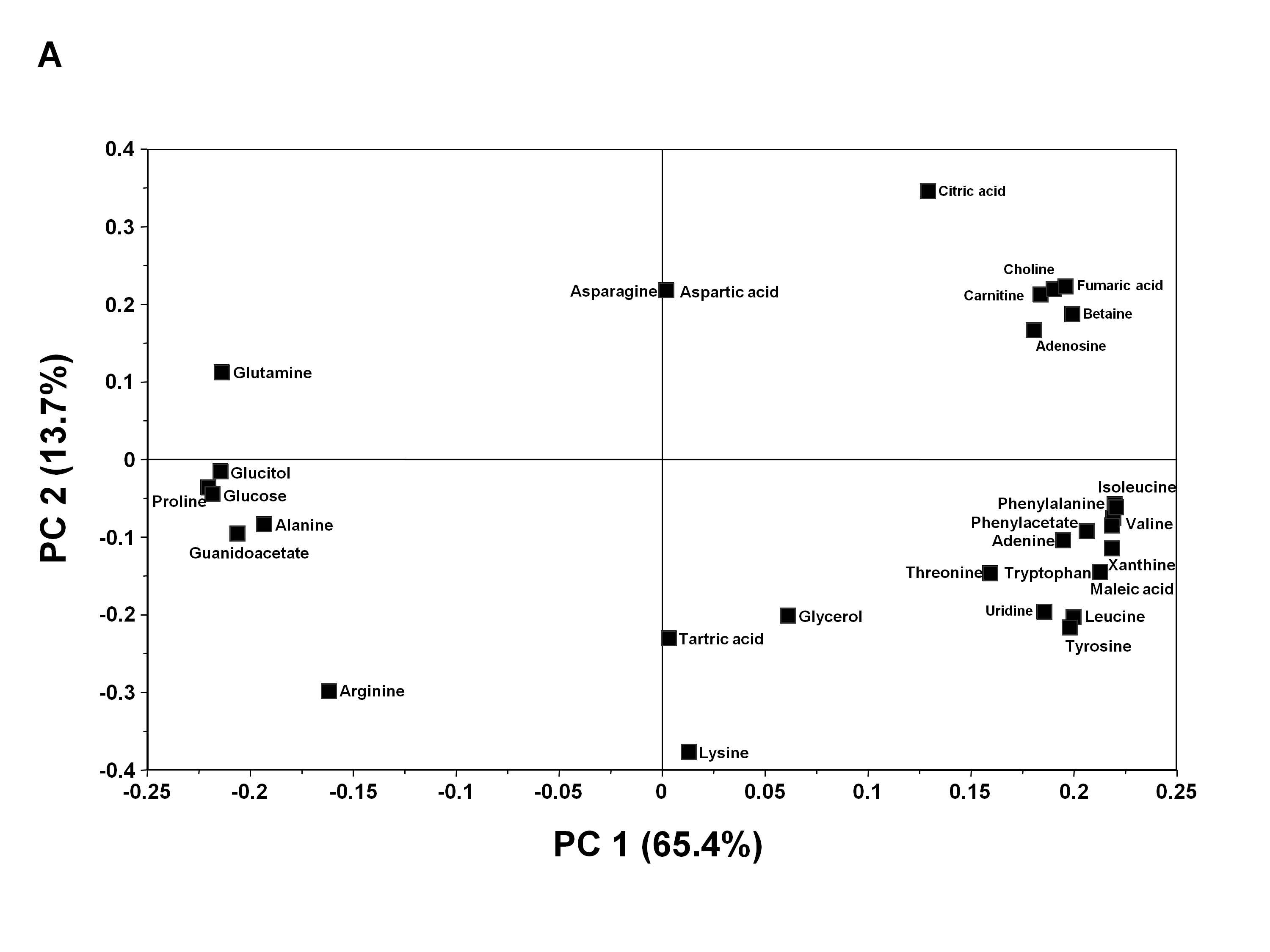

Supplement: Figure S2 — PCA loading plots derived from NMR analysis of D2O extracts of C. pruinosa mycelia cultivated under various conditions. (TIF) [file pone.0090823.s002.tif]

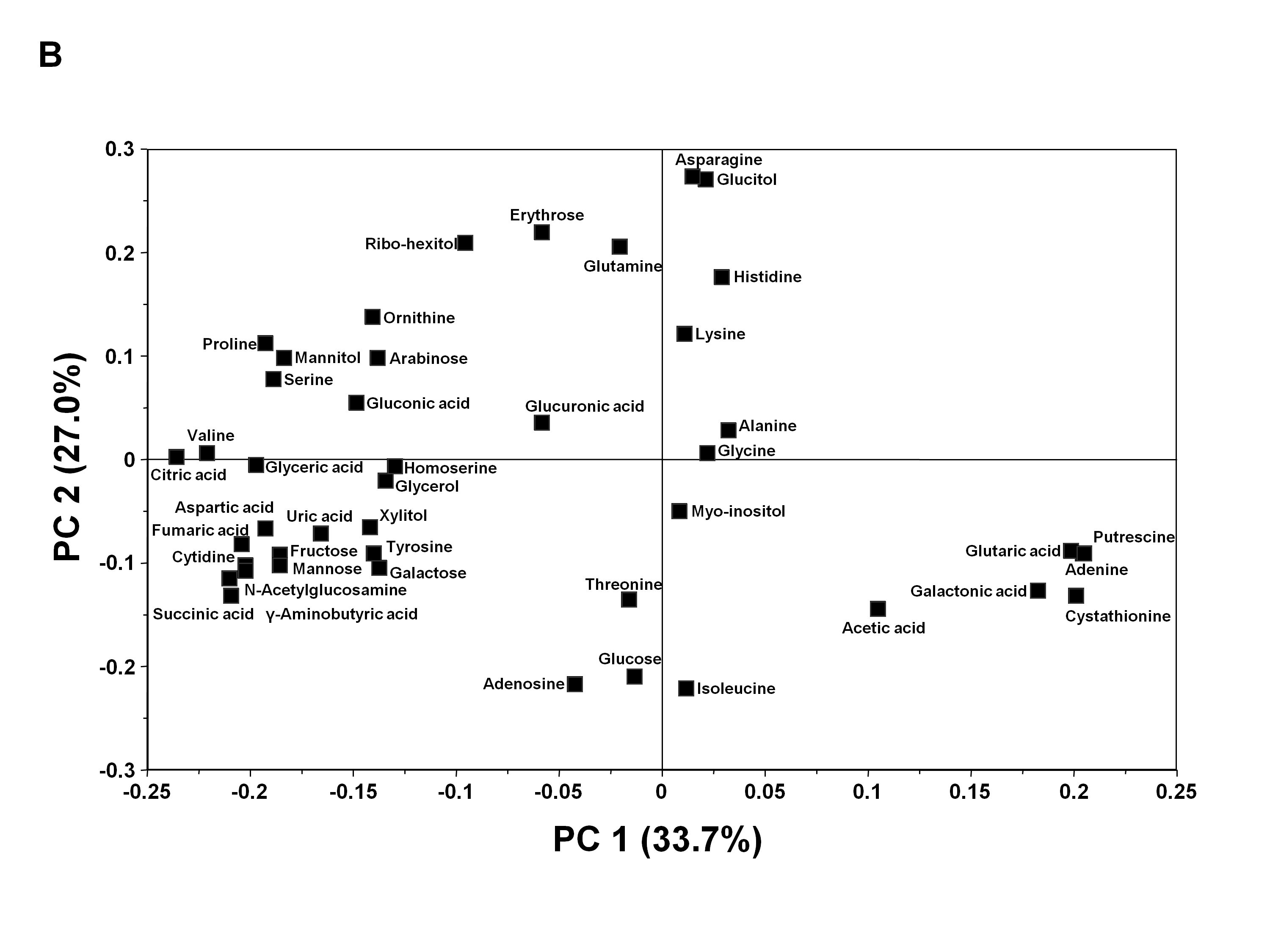

Supplement: Figure S3 — PCA loading plots derived from GC-MS analysis of 70% methanol extracts of C. pruinosa mycelia cultivated under various conditions. (TIF) [file pone.0090823.s003.tif]

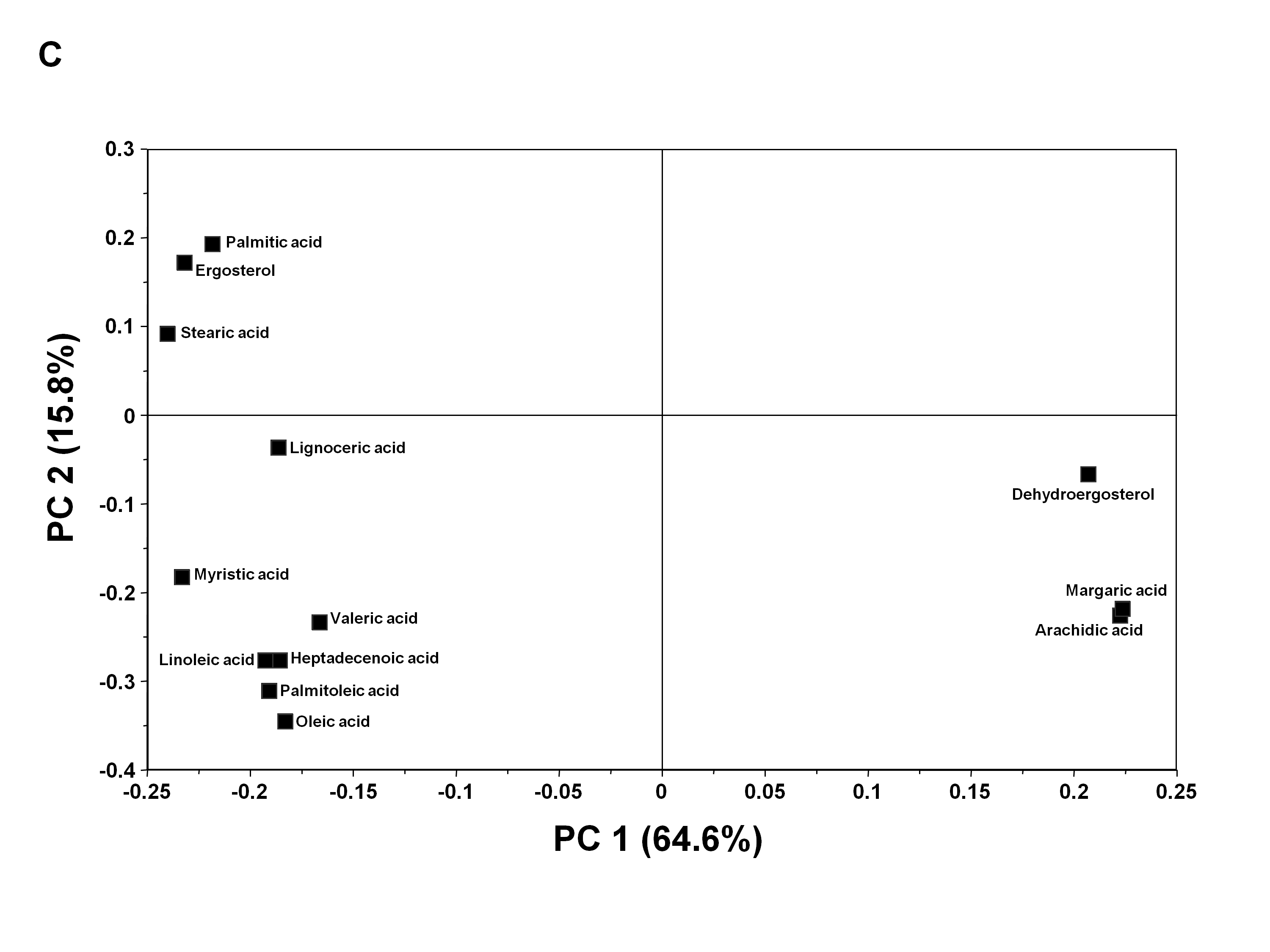

Supplement: Figure S4 — PCA loading plots derived from GC-MS analysis of 100% n -hexane extracts of C. pruinosa mycelia cultivated under various conditions. (TIF) [file pone.0090823.s004.tif]

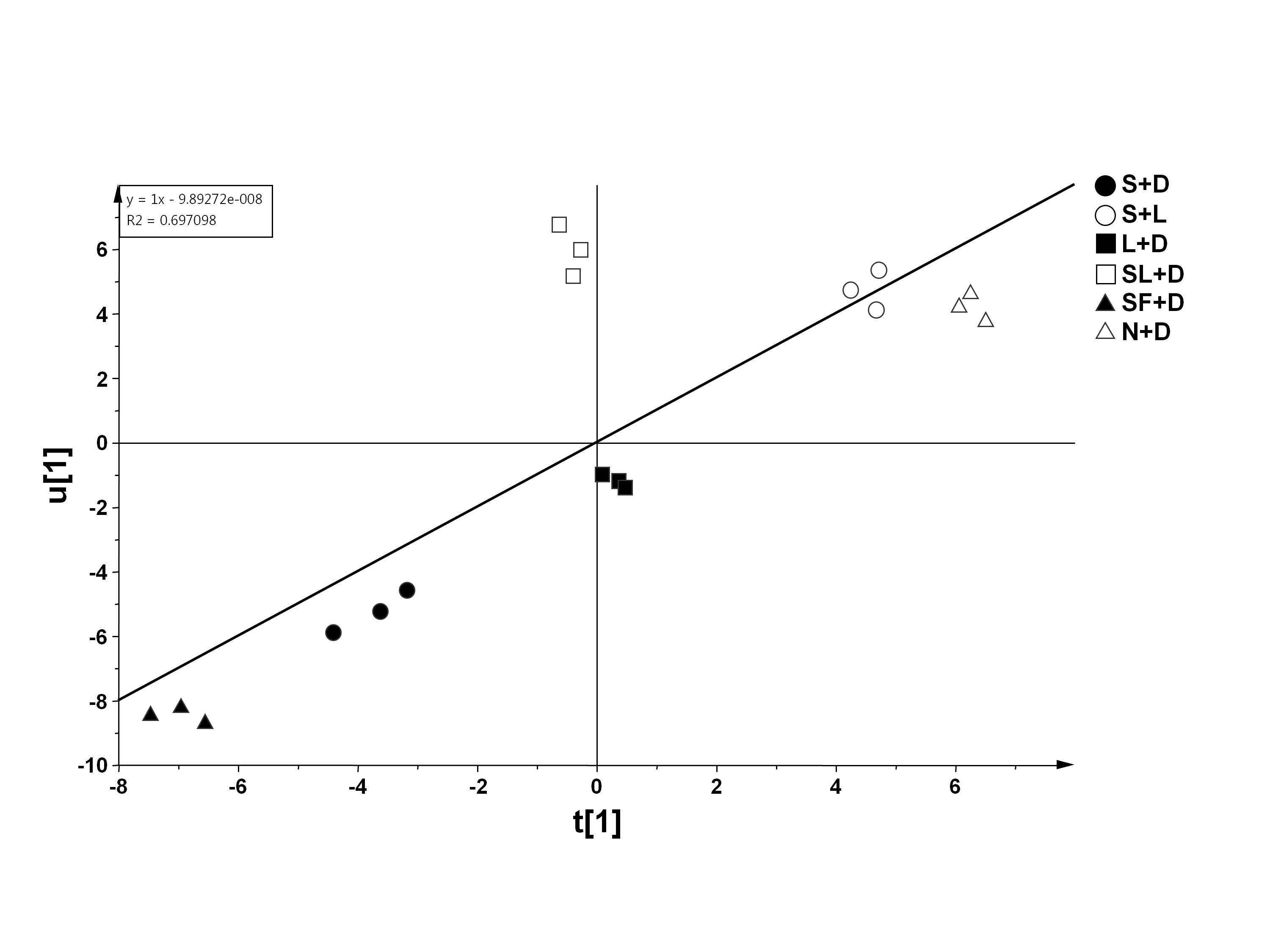

Supplement: Figure S5 — PLS t1/u1 score plots representing relationship between the free radical scavenging activities (u1) and metabolic profiles (t1) obtained by NMR and GC-MS. (TIF) [file pone.0090823.s005.tif]
