# Supplementary material for: NMR and GC-MS Based Metabolic Profiling and Free-Radical Scavenging Activities of Cordyceps pruinosa Mycelia Cultivated under Different Media and Light Conditions
Source: PLoS One. 2014 Mar 7;9(3):e90823. doi: 10.1371/journal.pone.0090823 (PMC3946585; doi:10.1371/journal.pone.0090823)
Supplement: Table S1 — A GC-MS-based metabolic profile of 70% methanol extracts of C. pruinosa mycelia. The relative levels of each metabolite were obtained by dividing the percentage area of each metabolite by the percentage area of the internal standard. Different letters in the same row indicate a significant difference. Mean±SD values for triplicate measurements are shown. ‘ND’ means ‘not detected’. (DOCX) [file pone.0090823.s006.docx]

**Table S1.** A GC-MS-based metabolic profile of 70% methanol extracts of *C. pruinosa* mycelia

The relative levels of each metabolite were obtained by dividing the percentage area of each metabolite by the percentage area of the internal standard. Different letters in the same row indicate a significant difference. Mean±SD values for triplicate measurements are shown. ‘ND’ means ‘not detected’.

| **Compound** | **RT**  **(min)** | **Relative intensity** | | | | | |
| --- | --- | --- | --- | --- | --- | --- | --- |
|  |  | **S+D** | **S+L** | **L+D** | **SL+D** | **SF+D** | **N+D** |
| **Alcohols** |  |  |  |  |  |  |  |
| Glucitol | 39.14 | ND | 0.04±0.002 | ND | ND | ND | ND |
| Glycerol | 13.14 | 1.13±0.05^ab^ | 1.15±0.05^ab^ | 0.48±0.02^a^ | 2.26±0.49^c^ | 1.75±0.002^bc^ | 0.92±0.04^ab^ |
| Myo-inositol | 32.03 | 1.17±0.04^a^ | 0.85±0.04^bc^ | 7.05±0.05^d^ | 2.73±0.09^e^ | 1.00±0.02^ac^ | 0.70±0.02^b^ |
|  | 33.46 |  |  |  |  |  |  |
|  | 34.61 |  |  |  |  |  |  |
|  | 34.75 |  |  |  |  |  |  |
|  | 40.05 |  |  |  |  |  |  |
| Mannitol | 31.53 | 19.52±0.86^a^ | 20.33±0.65^a^ | 4.24±0.49^b^ | 9.48±0.20^c^ | 25.73±1.04^d^ | 3.12±0.09^b^ |
| Ribo-hexitol | 29.56 | 0.84±0.04^ab^ | 1.10±0.06^c^ | 0.540±0.003^d^ | 0.91±0.03^b^ | 0.67±0.02^ad^ | 0.52±0.03^d^ |
| Xylitol | 26.71 | ND | ND | ND | ND | 0.65±0.02 | ND |
|  | 27.05 |  |  |  |  |  |  |
| **Amine** |  |  |  |  |  |  |  |
| Putrescine | 27.35 | ND | ND | ND | ND | ND | 0.22±0.01 |
| **Amino acids** |  |  |  |  |  |  |  |
| Alanine | 18.93 | 0.10±0.005^ab^ | 0.15±0.01^bc^ | 0.24±0.03^d^ | 0.21±0.01^cd^ | 0.08±0.001^a^ | 0.11±0.01^ab^ |
| Asparagine | 25.86 | 0.21±0.01^ab^ | 1.16±0.05^c^ | 0.33±0.02^d^ | 0.29±0.01^bd^ | 0.15±0.001^a^ | 0.14±0.004^a^ |
| Aspartic acid | 21.95 | 4.92±0.30^a^ | 3.67±0.16^b^ | 4.89±0.23^a^ | 5.83±0.22^c^ | 4.89±0.21^a^ | 2.67±0.10^d^ |
|  | 37.97 |  |  |  |  |  |  |
| Cystathionine | 36.86 | 1.01±0.07^ab^ | 0.82±0.02^a^ | 1.66±0.07^c^ | 1.15±0.02^b^ | 1.22±0.09^b^ | 3.49±0.05^d^ |
| Glutamine | 24.69 | 16.86±0.37^a^ | 18.45±0.71^a^ | 14.23±0.80^bc^ | 15.98±0.15^ac^ | 13.23±0.63^b^ | 14.00±0.11^bc^ |
|  | 28.32 |  |  |  |  |  |  |
| Glycine | 14.25 | 1.12±0.03^a^ | 1.40±0.08^bc^ | 1.82±0.04^d^ | 1.21±0.02^ab^ | 1.52±0.02^c^ | 1.34±0.03^bc^ |
|  | 20.31 |  |  |  |  |  |  |
| Histidine | 31.33 | 0.92±0.07^a^ | 1.41±0.08^b^ | 1.33±0.01^b^ | 1.20±0.02b^c^ | 1.10±0.02^ac^ | 1.07±0.02^ab^ |
| Homoserine | 19.72 | 0.10±0.005^a^ | 0.06±0.005^bc^ | 0.07±0.002^c^ | 0.10±0.002^a^ | 0.05±0.001^b^ | 0.04±0.002^d^ |
| Isoleucine | 13.82 | 0.39±0.02^ab^ | 0.24±0.01^c^ | 0.49±0.01^d^ | 0.36±0.005^a^ | 0.41±0.01^b^ | 0.41±0.01^b^ |
| Lysine | 26.80 | 3.45±0.14^ab^ | 4.58±0.23^c^ | 3.30±0.08^ad^ | 2.74±0.06^d^ | 4.53±0.09^c^ | 4.02±0.06^bc^ |
|  | 31.47 |  |  |  |  |  |  |
| Ornithine | 29.22 | 0.73±0.04^a^ | 1.56±0.04^b^ | 1.28±0.04^c^ | 0.64±0.01^a^ | 1.67±0.02^b^ | 0.19±0.01^d^ |
| Proline | 13.96 | 25.01±0.71^a^ | 27.85±1.16^a^ | 27.26±1.12^a^ | 26.95±0.10^a^ | 27.40±0.47^a^ | 14.64±0.27^b^ |
|  | 21.79 |  |  |  |  |  |  |
|  | 22.04 |  |  |  |  |  |  |
| Serine | 12.44 | 2.87±0.13^a^ | 2.79±0.09^a^ | 2.93±0.09^a^ | 2.80±0.07^a^ | 2.79±0.01^a^ | 1.95±0.02^b^ |
|  | 16.49 |  |  |  |  |  |  |
| Threonine | 17.46 | 2.41±0.08^a^ | 1.66±0.07^b^ | 2.49±0.04^a^ | 2.37±0.07^a^ | 1.79±0.02^bc^ | 1.98±0.04^c^ |
| Tyrosine | 31.74 | 1.24±0.06^abc^ | 0.80±0.02^ab^ | 1.81±0.11^c^ | 1.38±0.02^bc^ | 1.57±0.03^c^ | 0.63±0.31^a^ |
| Valine | 10.91 | 1.52±0.07^ab^ | 1.01±0.04^c^ | 1.43±0.04^ab^ | 1.35±0.03^a^ | 1.56±0.03^b^ | ND |
| **Organic acids** |  |  |  |  |  |  |  |
| Acetic acid | 19.21 | 1.80±0.43^ab^ | 0.30±0.01^c^ | 1.16±0.03^a^ | 2.36±0.03^b^ | 0.23±0.01^c^ | 2.41±0.04^b^ |
| Citric acid | 29.35 | 15.81±0.44^a^ | 10.07±0.47^b^ | 9.46±0.18^b^ | 12.13±0.30^c^ | 15.29±0.15^a^ | 3.21±0.10^d^ |
| Fumaric acid | 16.18 | 0.08±0.01^a^ | 0.05±0.003^b^ | 0.05±0.001^b^ | 0.07±0.002^a^ | 0.14±0.004^c^ | 0.04±0.001^b^ |
| Galactonic acid | 32.82 | ND | ND | 0.20±0.003^a^ | ND | 0.09±0.04^b^ | 0.31±0.01^c^ |
| Gluconic acid | 32.90 | 0.07±0.02a | 0.09±0.001^a^ | 0.04±0.002^a^ | 0.11±0.01^a^ | 0.11±0.04^a^ | 0.04±0.002^a^ |
|  | 40.16 |  |  |  |  |  |  |
| Glucuronic acid | 39.58 | ND | 0.05±0.01^a^ | 0.04±0.001^a^ | 0.12±0.02^b^ | 0.04±0.02^a^ | 0.02±0.002^a^ |
| Glutaric acid | 23.53 | ND | ND | ND | ND | ND | 0.43±0.07 |
| Glyceric acid | 15.29 | 0.04±0.00^ab^ | 0.03±0.00^a^ | 0.01±0.01^c^ | 0.07±0.003^d^ | 0.05±0.002^b^ | ND |
| Succinic acid | 14.71 | 0.04±0.01^a^ | 0.03±0.00^a^ | 0.02±0.00^a^ | 0.07±0.00^a^ | 0.05±0.00^a^ | 0.43±0.12^b^ |
|  | 20.99 |  |  |  |  |  |  |
| γ-aminobutyric acid | 9.41 | 5.28±0.35^a^ | 1.98±0.14^b^ | 3.35±0.11^c^ | 5.39±0.16^a^ | 7.45±0.23^d^ | 2.24±0.03^b^ |
|  | 22.11 |  |  |  |  |  |  |
| **Purines** |  |  |  |  |  |  |  |
| Adenine | 30.22 | ND | ND | ND | ND | ND | 0.10±0.002 |
| Adenosine | 42.65 | 0.18±0.01^ab^ | ND | 0.05±0.004^c^ | 0.15±0.01^a^ | 0.21±0.01^b^ | 0.20±0.004^b^ |
|  | 44.06 |  |  |  |  |  |  |
|  | 46.15 |  |  |  |  |  |  |
| Uric acid | 34.87 | 0.01±0.001^abc^ | 0.01±0.004^ab^ | 0.03±0.01^bc^ | 0.02±0.001^abc^ | 0.04±0.01^c^ | ND |
| Xanthine | 33.31 | ND | ND | ND | ND | 0.01±0.004 | ND |
| **Pyrimidine** |  |  |  |  |  |  |  |
| Cytidine | 40.54 | 0.05±0.004^a^ | ND | ND | 0.06±0.004^a^ | 0.12±0.01^b^ | ND |
| **Sugars** |  |  |  |  |  |  |  |
| Arabinose | 29.13 | 0.05±0.01^a^ | 0.05±0.03^a^ | ND | 0.04±0.001^a^ | 0.05±0.03^a^ | ND |
| Erythrose | 21.57 | ND | 0.56±0.04^a^ | ND | ND | 0.33±0.04^b^ | ND |
| Fructose | 26.07 | 0.21±0.02^a^ | ND | ND | 0.05±0.001^b^ | 0.29±0.01^c^ | ND |
|  | 30.58 |  |  |  |  |  |  |
| Galactose | 29.82 | 0.18±0.03^a^ | 0.15±0.01^a^ | 0.26±0.02^ab^ | 0.28±0.07^ab^ | 0.43±0.08^b^ | 0.17±0.002^a^ |
|  | 30.66 |  |  |  |  |  |  |
|  | 31.00 |  |  |  |  |  |  |
|  | 39.44 |  |  |  |  |  |  |
|  | 41.19 |  |  |  |  |  |  |
| Glucose | 29.00 | 121.01±6.34^ab^ | 80.02±16.10^a^ | 126.62±15.22^ab^ | 142.99±13.07^b^ | 145.30±3.63^b^ | 144.27±6.40^b^ |
|  | 31.24 |  |  |  |  |  |  |
|  | 32.57 |  |  |  |  |  |  |
|  | 34.74 |  |  |  |  |  |  |
|  | 36.80 |  |  |  |  |  |  |
|  | 39.74 |  |  |  |  |  |  |
|  | 43.69 |  |  |  |  |  |  |
|  | 44.78 |  |  |  |  |  |  |
| Mannose | 30.83 | 2.62±0.14^a^ | 0.08±0.03^b^ | 0.52±0.01^c^ | 0.77±0.04^c^ | 4.25±0.02^d^ | 0.17±0.01^b^ |
| N-acetylglucosamine | 33.86 | 0.07±0.005^a^ | ND | ND | 0.05±0.002^b^ | 0.07±0.003^a^ | ND |
|  | 35.10 |  |  |  |  |  |  |
