# Supplementary material for: NMR and GC-MS Based Metabolic Profiling and Free-Radical Scavenging Activities of Cordyceps pruinosa Mycelia Cultivated under Different Media and Light Conditions
Source: PLoS One. 2014 Mar 7;9(3):e90823. doi: 10.1371/journal.pone.0090823 (PMC3946585; doi:10.1371/journal.pone.0090823)
Supplement: Table S2 — A GC-MS-based metabolic profile of 100% n-hexane extracts of C. pruinosa mycelia. The relative levels of each metabolite were obtained by dividing the percentage area of each metabolite by the percentage area of the internal standard. Different letters in the same row indicate a significant difference. Mean±SD values for triplicate measurements are shown. ‘ND’ means ‘not detected’. (DOCX) [file pone.0090823.s007.docx]

**Table S2.** A GC-MS-based metabolic profile of 100% n-hexane extracts of *C. pruinosa* mycelia

The relative levels of each metabolite were obtained by dividing the percentage area of each metabolite by the percentage area of the internal standard. Different letters in the same row indicate a significant difference. Mean±SD values for triplicate measurements are shown. ’ND’ means ‘not detected’.

| **Compound** | **RT**  **(min)** | **Relative intensity** | | | | | |
| --- | --- | --- | --- | --- | --- | --- | --- |
|  |  | **S+D** | **S+L** | **L+D** | **SL+D** | **SF+D** | **N+D** |
| **Pyrimidine** |  |  |  |  |  |  |  |
| Uracil | 11.70 | 0.60±0.04^a^ | 0.61±0.05^a^ | 0.59±0.03^a^ | 0.49±0.02^a^ | 0.47±0.03^a^ | ND |
| **Saturated fatty acids** |  |  |  |  |  |  |  |
| Myristic acid | 23.45 | 0.76±0.05^a^ | 1.47±0.16^b^ | 0.56±0.01^a^ | 0.66±0.03^a^ | 0.77±0.06^a^ | ND |
| Valeric acid | 25.42 | 0.68±0.06 ^a^ | 1.35±0.11^b^ | 0.80±0.09^ab^ | 0.92±0.04^ab^ | 0.92±0.03^ab^ | 0.56±0.28^a^ |
| Arachidic acid | 33.72 | ND | 0.32±0.03^a^ | ND | 0.19±0.10^a^ | 0.19±0.10^a^ | 2.89±0.17^b^ |
| Lignoceric acid | 39.30 | 0.18±0.09^a^ | 0.51±0.01^b^ | 0.29±0.06^ab^ | 0.35±0.07^ab^ | 0.51±0.05^b^ | ND |
| Margaric acid | 29.12 | 0.26±0.03^a^ | 0.55±0.05^ab^ | 0.38±0.04^ab^ | 0.60±0.03^b^ | 0.27±0.01^a^ | 3.02±0.15^c^ |
| Palmitic acid | 36.41 | 29.59±3.83^a^ | 22.22±2.34^a^ | 21.00±1.53^a^ | 20.79±1.60^a^ | 23.13±2.48^a^ | ND |
| Stearic acid | 30.89 | 34.08±2.63^a^ | 43.62±3.19^a^ | 38.68±1.85^a^ | 36.80±1.73^a^ | 36.44±1.74^a^ | 1.25±0.16^b^ |
| **Unsaturated fatty acids** |  |  |  |  |  |  |  |
| Heptadecenoic acid | 28.67 | 0.28±0.04^a^ | 0.68±0.09^b^ | ND | 0.32±0.04^a^ | 0.19±0.10^a^ | ND |
| Linoleic acid | 29.42 | 19.36±1.86^ab^ | 48.51±4.49^c^ | 26.03±2.39^b^ | 27.30±2.17^b^ | 18.74±0.25^ab^ | 11.11±0.87^a^ |
|  | 29.92 |  |  |  |  |  |  |
|  | 30.30 |  |  |  |  |  |  |
|  | 31.22 |  |  |  |  |  |  |
|  | 31.91 |  |  |  |  |  |  |
| Oleic acid | 29.54 | 25.22±3.49^a^ | 84.31±7.16^b^ | 17.46±1.63^ac^ | 25.59±2.10^a^ | 27.04±1.24^a^ | 8.20±0.16^c^ |
|  | 30.42 |  |  |  |  |  |  |
|  | 30.50 |  |  |  |  |  |  |
|  | 30.60 |  |  |  |  |  |  |
|  | 38.45 |  |  |  |  |  |  |
| Palmitoleic acid | 26.91 | 4.67±0.60^a^ | 11.79±1.26^b^ | 0.47±0.04^c^ | 2.60±0.11^ac^ | 4.18±0.18^a^ | ND |
| **Sterols** |  |  |  |  |  |  |  |
| Dehydroergosterol | 43.66 | ND | ND | 0.34±0.03^a^ | ND | ND | 0.49±0.03^b^ |
| Ergosterol | 43.41 | 29.73±1.27^a^ | 27.35±3.06^a^ | 27.69±2.59^a^ | 27.95±1.31^a^ | 25.56±1.62^a^ | 4.25±0.04^b^ |
